# Legumain in cardiovascular diseases

**DOI:** 10.3389/ebm.2024.10121

**Published:** 2024-07-22

**Authors:** Lei Zhou, Jianqiang Wu, Zairong Wei, Yuehong Zheng

**Affiliations:** ^1^ Department of Burns and Plastic Surgery, Affiliated Hospital of Zunyi Medical University, Zunyi, China; ^2^ Department of Vascular Surgery, State Key Laboratory of Complex Severe and Rare Diseases, Peking Union Medical College Hospital, Chinese Academy of Medical Sciences and Peking Union Medical College, Beijing, China; ^3^ Institute of Clinical Medicine, National Science and Technology Key Infrastructure on Translational Medicine, State Key Laboratory of Complex Severe and Rare Diseases, Peking Union Medical College Hospital, Chinese Academy of Medical Sciences and Peking Union Medical College, Beijing, China

**Keywords:** legumain, cardiovascular disease, atherosclerosis, coronary artery disease, aortic disease

## Abstract

Cardiovascular diseases (CVDs) are the leading cause of death worldwide, having become a global public health problem, so the pathophysiological mechanisms and therapeutic strategies of CVDs need further study. Legumain is a powerful enzyme that is widely distributed in mammals and plays an important role in a variety of biological processes. Recent research suggests that legumain is associated with the occurrence and progression of CVDs. In this review, we provide a comprehensive overview of legumain in the pathogenesis of CVDs. The role of legumain in CVDs, such as carotid atherosclerosis, pulmonary hypertension, coronary artery disease, peripheral arterial disease, aortic aneurysms and dissection, is discussed. The potential applications of legumain as a biomarker of these diseases are also explored. By understanding the role of legumain in the pathogenesis of CVDs, we aim to support new therapeutic strategies to prevent or treat these diseases.

## Impact statement

CVDs are the leading cause of death globally and remain a heavy unresolved societal burden. The discovery of novel biomarkers and therapeutic targets may improve the situation. Legumain is involved in the occurrence, development, and prognosis of multiple CVDs. This work details the action mechanism of legumain in CVDs to develop new therapeutic targets. It also describes the research progress on legumain as a biomarker for CVDs.

## Introduction

Cardiovascular diseases (CVDs) are the leading cause of death worldwide [1]. In recent years, with the continuous development of biomarkers and new drugs, the secondary prevention of CVDs has made significant progress, but their mortality rate is still high. Therefore, it is still necessary to further explore the pathogenesis of CVDs and find new strategies for diagnosis and treatment.

Cysteine proteases are involved in the degradation of intracellular proteins and the extracellular matrix (ECM), protein processing and cell signal transduction, playing an important role in the pathogenesis of CVDs, and their inhibitors have therapeutic potential for CVDs [2]. As a member of the cysteine protease family, legumain (LGMN) augments the occurrence and development of atherosclerosis, pulmonary hypertension, peripheral vascular disease, aortic aneurysm and dissection, and promotes the outcome of coronary heart disease. In this review, we discuss mechanistic and biomarker-related studies of LGMN in CVDs and explore LGMN as a potential therapeutic target for these diseases.

## The origin and regulation of LGMN

### Source of LGMN

LGMN, also known as asparaginyl endopeptidase (AEP), belongs to the C13 family of cysteine proteases. It was first identified in the early 1980s and received its name in 1993 for its function in legume seeds [3, 4]. In 1996, LGMN was identified in mammalian tissues, and it is widely distributed in various tissues, including the testes, kidneys, liver, heart, and vasculature [5, 6].

In cells, mature LGMN is located primarily in the lysosome. Prolegumain is translocated from the endoplasmic reticulum and Golgi apparatus to the lysosomal system and activated in the acidic environment of the lysosome. Prolegumain can be secreted extracellularly directly through the Golgi apparatus or via the lysosomal/endosomal system [7], allowing LGMN to be detected in the plasma/serum of patients.

### Activation of LGMN

Prolegumain has 433 amino acids and molecular mass of 56 kDa. It has 3 parts: the catalytic domain, activation peptide (AP) and LGMN stabilization and activity-modulating (LSAM) domain. Prolegumain has no protease activity because the LSAM covers the catalytic active site. In an acidic environment, LGMN undergoes a pH-dependent conformational change, and at pH < 4.5, autocatalytic cleavage exposes the active site (Cys189) in the catalytic domain to yield biologically active mature LGMN with a molecular mass of 36 kDa [8, 9]. LGMN is activated primarily in lysosomes and can be delivered extracellularly. According to the latest literature, the secreted protein LGMN can act as a novel signaling molecule to perform biological functions outside the cells [10, 11].

### Inducers and inhibitors of LGMN

Hypoxia-inducible factor 1-alpha (HIF1α) is a nuclear transcription factor that directly increases the gene and protein expression levels of LGMN [11, 12]. *In vitro* studies revealed that primary human monocytes polarized to M1 macrophages in response to the proinflammatory factors lipopolysaccharide (LPS) and IFN-γ had 9-fold higher LGMN protein expression levels than IL-4-polarized M2 macrophages, whereas no significant difference in LGMN secretion was observed between M2-type and resting macrophages [13]. Various proinflammatory cytokines, such as Interleukin-1β (IL-1β), interferon-γ (IFN-γ), and tumor necrosis factor-α (TNF-α), can increase the protein expression levels of various cysteine proteases in monocytes and vascular cells [14–16]. The protein expression levels of LGMN may also be regulated by these cytokines and further research is needed.

Currently, several small-molecule substrates, inhibitors, and activity-based probes of LGMN have been developed [17]. The inhibitor RR-11a appears to be a promising agent for disease treatment. RR-11a is a synthetic LGMN-specific inhibitor that irreversibly inhibits LGMN function by forming a covalent bond to the catalytic cysteine Cys189, demonstrating potential therapeutic utility in several CVD disease models [10, 18, 19].

## LGMN in CVDs

### Carotid atherosclerosis

Atherosclerosis is a common CVD characterized by the accumulation of lipids and other molecules in the walls of arteries and the formation of plaques, resulting in the narrowing and hardening of blood vessels, such as medium and large arteries, throughout the body [20, 21]. LGMN plays a key role in the development of atherosclerosis. LGMN was increased in the plasma and arterial tissue of patients with carotid atherosclerosis as well as in mouse models of atherosclerosis [22, 23]. Carotid artery stenosis, i.e., atherosclerotic narrowing or even occlusion of the carotid arteries, can produce serious or lethal neurological consequences, so identifying potential diagnostic, therapeutic and risk stratification methods for carotid artery stenosis is essential [24]. LGMN protein expression levels are elevated in the plasma and plaques of patients with carotid stenosis [15], suggesting that it may be involved in the occurrence and progression of this disease.

The mechanism of action of LGMN in atherosclerosis is complex. LGMN can indirectly participate in the degradation of the ECM by activating cathepsins B, H, and L, as well as matrix metalloproteinase-2 (MMP-2) [25, 26], enzymes that promote atherogenesis by degrading ECM collagen and elastin [27–29]. LGMN can directly degrade fibronectin in the ECM [30]. Degradation of the ECM allows the accumulation of lipids and other substances, and facilitates the formation of fatty streaks in the arterial wall, the earliest stage of atherosclerosis. In addition, the protein expression levels of LGMN, MMPs, and cathepsins are higher in unstable than stable plaques. A potential activator of MMPs and cathepsins, LGMN may destabilize plaques by activating these ECM-degrading enzymes.

LGMN is involved in tumor neovascularization [31, 32]. In atherosclerotic plaques, intraplaque angiogenesis enhances deposition but also fragility and instability of plaques [33]. Further studies are necessary to understand the mechanisms of LGMN in plaque stability and to develop effective therapeutic strategies. The increased protein expression levels of LGMN in unstable plaques could be used to identify people at high risk of developing CVDs, allowing for early detection and preventive treatment.

The protein expression level of LGMN is elevated in macrophages of atherosclerosis. LGMN expression, activity and secretion are significantly enhanced during the differentiation of monocytes to macrophages, the major inflammatory cells in atherosclerosis. The protein expression level of LGMN is higher in proinflammatory M1 macrophages than in anti-inflammatory M2 macrophages. These findings suggest that LGMN may regulate monocyte/macrophage inflammatory functions [15, 34]. *In vitro* migration assays revealed that LGMN dose-dependently increased the migration of monocytes and their adhesion to endothelial cells [23], followed by their migration in the subendothelium, where they differentiated into macrophages, initiating the formation of atherosclerotic lesions [35]. LGMN can be secreted extracellularly, and the chemotactic effect of extracellular LGMN on monocytes may contribute to the recruitment of monocytes/macrophages into atherosclerotic lesions and exacerbate inflammation in the vessel wall. *In vitro*, LGMN increases endothelial cell expression of proinflammatory cytokines, such as monocyte chemoattractant protein-1 (MCP-1) [5], which is a multifunctional chemokine that promotes monocyte adhesion, migration, and induction of inflammatory responses.

Foam cells, macrophages that phagocytose large amounts of lipid, are atherosclerotic hallmarks. Stimulation with cholesterol crystals significantly increases the secretion of LGMN by M1-type macrophages, suggesting that LGMN may promote lipid metabolism [15]. LGMN increases the protein expression levels of scavenger receptor class A (SR-A), acyl-coenzyme A: cholesterol acyltransferase-1 (ACAT-1), and neutral cholesterol ester hydrolase (NCEH) in macrophages and may regulate lipid metabolism through this pathway to increase oxidized low-density lipoprotein (oxLDL) induced foam cell formation [5]. SR-A is one of the main receptors responsible for ox-LDL binding and uptake in macrophages [36]. Uncontrolled uptake of ox-LDL leads to impaired intracellular cholesterol metabolism and the accumulation of cytoplasmic lipid droplets, subsequently triggering the formation of foam cells. ACAT1 and NCEH play key roles in cholesterol esterification. ACAT1 esterifies free cholesterol in the endoplasmic reticulum to form cholesteryl esters. NCEH can hydrolyze lipid droplets in the cytoplasm and excrete them from the cell [37]. The balance between ACAT1 and NCEH regulates intracellular lipid metabolism.

The discovery, validation and implementation of novel biomarkers are important for clarifying disease diagnoses, determining disease severity and predicting clinical prognoses. The protein expression levels of LGMN was significantly higher in the plasma and plaques of patients with carotid stenosis than in healthy control individuals and was increased in the carotid plaques of recently symptomatic patients compared to asymptomatic patients. These findings suggest that the protein expression levels of LGMN is elevated in stenotic disease and indicate that LGMN may be a useful atherosclerotic biomarker for diagnosing and assessing the disease [15]. LGMN protein promotes plaque instability, further suggesting that LGMN may be a new therapeutic target for unstable plaques and a biomarker that can predict clinical prognosis to diagnose and assess disease [22].

### Peripheral artery disease

Peripheral artery disease (PAD) affects more than 200 million people worldwide and is most commonly caused by atherosclerosis. Atherosclerotic plaques cause arterial stenosis which limits blood supply to distal tissues [38, 39]. Most patients with PAD have no obvious symptoms. When stenosis is severe, it can cause intermittent claudication, resting pain, and even foot ulcers and gangrene, which not only seriously affect the patient’s quality of life but may also lead to amputation or even death [39]. Serum LGMN was significantly higher in PAD patients than in non-PAD patients, and high serum LGMN was independently associated with an increased risk of PAD [40]. These findings suggest LGMN could be a blood biomarker and predictor of PAD. The mechanism of action of LGMN and its roles as a therapeutic target and biomarker of atherosclerosis-related diseases deserve further study.

### Pulmonary arterial hypertension

Pulmonary arterial hypertension (PAH) is a serious and potentially life-threatening condition characterized by increased vascular stiffness, which can lead to severe symptoms such as progressive dyspnea, fatigue, chest pain, and syncope [41].

LGMN serum concentration paralleled the severity of PAH, suggesting that this protein may be involved in disease progression [19]. LGMN knockdown or inhibition reduces the severity of PAH in animal models. In PAH, elastin degradation, collagen deposition and cross-linking, and tenascin and fibronectin deposition in the ECM lead to pulmonary vascular wall remodeling and vascular stiffening [42]. The transforming growth factor-β1 (TGF-β1) signaling pathway is a key initiator and driver of ECM protein deposition and fibrotic disease [43, 44], and excessive TGF-β-mediated signaling promotes PAH development [45]. Macrophage-derived LGMN promotes the deposition of collagen I, fibronectin and tenascin-C in the ECM via the MMP2/TGF-β signaling pathway, contributing to the progression of PAH [19]. LGMN promotes the synthesis of ECM components in PAH and the degradation of ECM components in atherosclerosis, further demonstrating the key role of LGMN in tissue remodeling.

The involvement of LGMN in pulmonary vascular wall remodeling has implications for the occurrence and progression of PAH. Targeting LGMN might reduce pulmonary vascular wall remodeling to improve symptoms. In addition, the LGMN inhibitor RR-11a may be used to reduce the risk and progression of PAH [19]. Further studies are needed to fully understand the role of LGMN in the development of PAH and to develop safe and effective treatments.

### Coronary artery disease

The global epidemic of coronary artery disease (CAD) is imposing ever-increasing rates of morbidity and mortality [46]. CAD is caused by atherosclerotic plaque accumulation leading to coronary artery stenosis or occlusion, and acute attacks may lead to conditions such as myocardial infarction and angina pectoris. LGMN was overexpressed in the plasma and tissues of acute CAD patients, suggesting that it may play an important role in the occurrence and progression of this disease [18, 47–49]. One study also found plasma LGMN to be independently associated with complex CAD [49], suggesting that LGMN may be a potential biomarker of CAD. Plasma LGMN levels have also been associated with patient outcomes. A study of patients with acute coronary syndrome with a 1-month follow-up showed that plasma LGMN was negatively associated with the incidence of stroke [47]. However, two studies with long-term follow-up of all-cause mortality in the myocardial infarction population yielded opposite results: One showed that low plasma LGMN was associated with increased mortality in the myocardial infarction population [48]. The other showed that all-cause mortality was significantly higher in the high-plasma-LGMN group than in the low-LGMN group [18]. Thus, prospective studies with larger cohorts are needed to clarify the predictive role of LGMN in acute myocardial infarction patients.

A recent study has shown that LGMN colocalized with platelets in arterial plaques and was released in response to platelet activation. Circulating LGMN protein was significantly correlated with plasma levels of the platelet activation markers P-selectin and platelet factor 4 (PF4), further suggesting that LGMN plays an important role in plaque stability and thrombosis [48]. Circulating LGMN is elevated in patients with acute myocardial infarction, a potentially deadly manifestation of CAD [18, 48]. In animal studies, the LGMN inhibitor RR-11a was found to improve myocardial remodeling and reduce the rate of cardiac rupture by inhibiting ECM degradation, demonstrating that LGMN may be a therapeutic target for myocardial infarction.

LGMN is also involved in regulating the efferocytosis of apoptotic cells. Efferocytosis is the phagocytosis and removal of cells that have undergone programmed cell death by macrophages. The timely removal of dead cells is essential to maintain physiological homeostasis or pathological improvements and to avoid further damage to surrounding tissues by the release of intracellular contents from dead cells [50, 51]. Macrophage secretion of LGMN was required to promote the efferocytosis of apoptotic cardiomyocytes in myocardial infarction, and selective overexpression of LGMN by macrophages improved cardiac function in mice after myocardial infarction. LGMN knockout resulted in the accumulation of apoptotic cardiomyocytes and exacerbated myocardial infarction [52].

The development and prognosis of myocardial infarction can be improved by regulating the function of LGMN, which will require more studies to clarify the function of LGMN in disease occurrence, progression, and recovery.

### Aortic aneurysm and aortic dissection

Aortic aneurysms and dissection are severely life-threatening, and mortality after aortic rupture can be as high as 85% [53]. The formation of aortic aneurysm and dissection may be related to ECM degradation, vessel wall inflammation, oxidative stress, and vascular smooth muscle cell (VSMC) dysfunction [54, 55]. The exact mechanism of aortic aneurysm and dissection is unknown, but recent studies have identified increased the protein expression levels of LGMN in the plasma and tissue of patients with aortic aneurysm and dissection [10, 56] and have identified a new potential role for LGMN in the pathogenesis of aortic aneurysm and dissection.

LGMN promotes degradation of the ECM components of the aortic wall and alters the structure and integrity of the aortic wall, which may be related to the formation of aortic aneurysms and dissection. Recent studies have shown that LGMN promoted the formation of thoracic aortic dissection by participating in the phenotypic switching of VSMCs [10]. VSMCs in healthy vessel walls exhibited a contractile phenotype to maintain vascular tone, moderating blood pressure and flow through their contractile and diastolic capacities [57]. The loss of VSMC contractile function altered vascular tone, increased aortic wall stress, and decreased vascular elasticity, thereby promoting aneurysm and dissection formation [54]. During thoracic aortic dissection, LGMN binds and blocks integrin αvβ3, thereby attenuating Rho GTPase activation, downregulating VSMC differentiation markers [10]. This study also showed that LGMN deficiency or inhibition can inhibit ECM degradation and VSMC transformation to synthetic type, reducing the incidence, mortality and degree of aortic dilatation in animal models. The results show that LGMN may be a potential therapeutic target for thoracic aortic dissection.

No diagnostic biomarkers or therapeutic agents are approved for abdominal aortic aneurysm (AAA). A recent study identified potential diagnostic biomarkers through proteomics analysis of AAA patients and healthy control individuals [56]. It identified 39 differentially expressed plasma proteins and subjected them to ROC analysis. LGMN was screened as a key protein that could be used as a diagnostic biomarker for AAA and further validated by ELISA in a larger cohort. LGMN may become a novel biomarker or therapeutic target for AAA [56].

## Discussion

**FIGURE 1:**
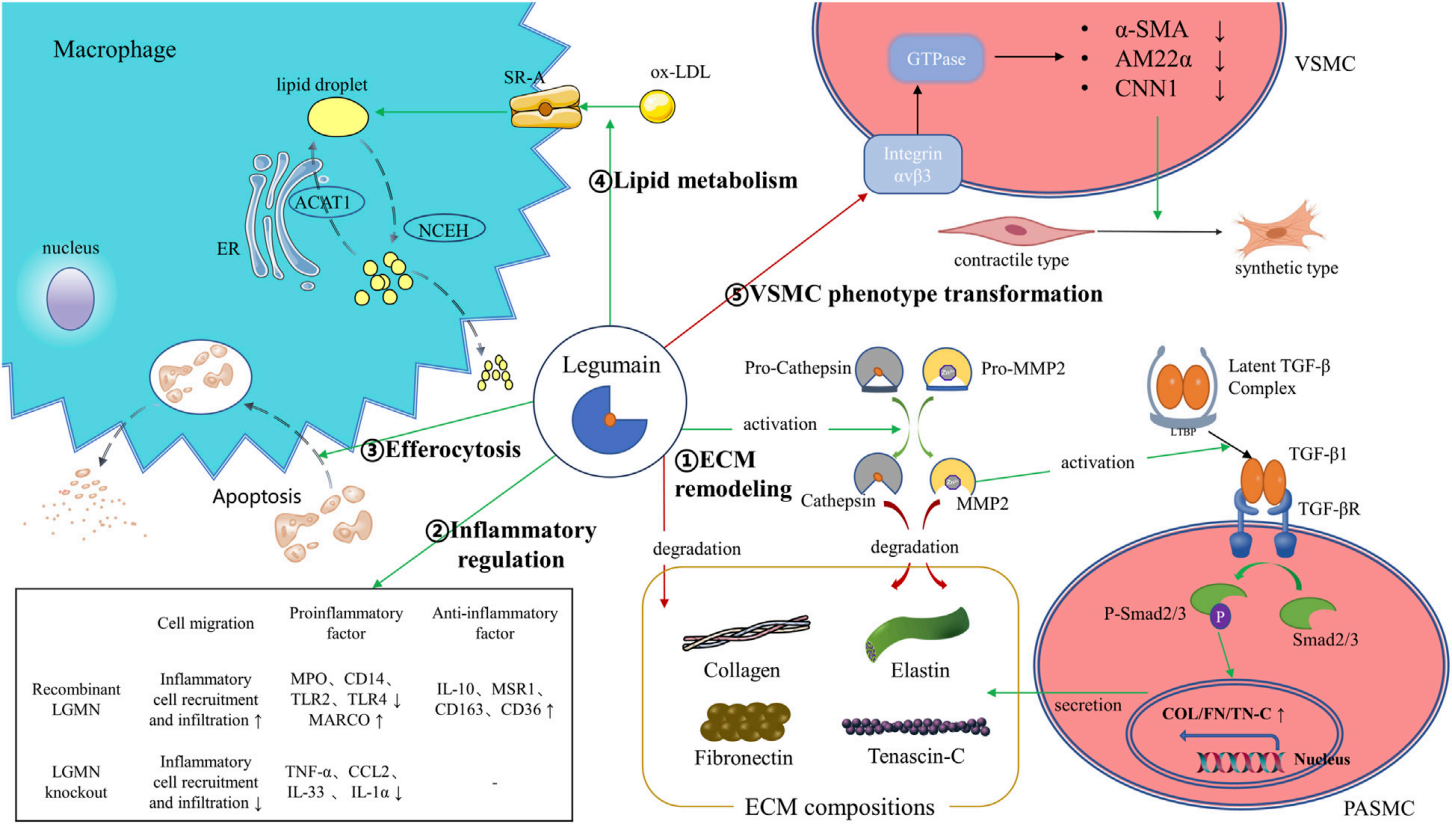
Illustration of the legumain pathway. ①LGMN could promote ECM degradation by activation of proMMP-2, processing of cathepsins or by direct proteolysis of ECM components like fibronectin. Meanwhile, LGMN promotes ECM remodeling by activating the MMP-2/TGF-β1 signaling. ②LGMN is involved in regulating the recruitment and infiltration of inflammatory cells, as well as regulating the expression of intracellular inflammatory factors. ③LGMN promotes clearance and degradation of apoptotic cardiomyocytes by efferocytosis. ④LGMN promotes macrophage uptake of ox-LDL to form lipid droplets by increasing SR-A expression. ⑤LGMN binds and blocks integrin αvβ3, thereby attenuating Rho GTPase activation and downregulating VSMC differentiation markers. ER: endoplasmic reticulum; ACAT1: acyl-coenzyme A: cholesterol acyltransferase-1; NCEH: neutral cholesterol ester hydrolase; SR-A: scavenger receptor class A; ox-LDL: oxidized low-density lipoprotein; α-SMA: α–smooth muscle actin; AM22α: smooth muscle 22α; CNN1: calponin 1; TGF-β: transforming growth factor-β; PASMC: pulmonary artery smooth muscle cell; MARCO: macrophage receptor with collagenous structure; IL: Interleukin; MPO: myeloperoxidase.

Current studies indicate that LGMN may serve as a potential plasma biomarker for the diagnosis, severity, and prognosis of various CVDs (Table 1). Because the sensitivity and specificity of a single biomarker are often inadequate [58], the establishment of diagnostic models combining LGMN with other differentially expressed proteins or traditional biochemical indicators will further improve the diagnostic performance of LGMN for CVDs. Meanwhile, recent evidence suggests that LGMN is a multifunctional protein. In addition to LGMN’s well-known lysosomal protease activity, its protease-independent functions have also been increasingly recognized, including regulation of inflammation, ECM remodeling, lipid metabolism, efferocytosis, and VSMC phenotypic transformation in CVDs (Figure 1). Therefore, LGMN is a promising therapeutic target for CVDs.

**TABLE 1:** Legumain has potential as a biomarker or therapeutic target for CVDs.

Study	Disease(s)	Result
Papaspyridonos M *et al.*, 2006	unstable atherosclerotic plaque	LGMN may be a novel target for the treatment of unstable plaque or useful diagnostic markers of plaque instability
Lunde NN *et al.*, 2017	atherosclerosis	LGMN was increased in both plasma and plaques of patients with carotid stenosis and might be a new and early biomarker of atherosclerosis
Bai P *et al.*, 2019	pulmonary hypertension	LGMN may serve as a valuable biomarker for evaluating the efficiency of PAH treatment
Fang Y *et al.*, 2019	atherosclerosis	LGMN can be used as a reference marker of atherosclerosis
Lunde NN *et al.*, 2020	acute cardiovascular events	LGMN was upregulated during acute cardiovascular events and was associated with improved outcome
Umei TC *et al.*, 2020	complex coronary lesions	Plasma LGMN level could become a biomarker for complex coronary lesions. High legumain levels in patients with CAD reflected coronary plaque instability
Yang H *et al.*, 2020	acute myocardial infarction	LGMN was not only a serological biomarker for inflammation and cardiac remodeling but also a pathogenic element responsible for the poor prognosis of AMI patients
Wei W *et al.*, 2021	peripheral artery disease	High serum LGMN was correlated with increased risk of PAD in T2DM patients
Wu J *et al.*, 2022	abdominal aortic aneurysm	LGMN is a novel biomarker of AAA with high diagnostic performance
He Y *et al.*, 2024	hypertension	Specifically targeting LGMN in Tregs may be an innovative approach for hypertension treatment

LGMN: legumain; PAH: pulmonary arterial hypertension; CAD: coronary artery disease; AMI: acute myocardial infarction; PAD: peripheral artery disease; T2DM: type 2 diabetes mellitus, AAA: abdominal aortic aneurysm.

LGMN knockout inhibits the upregulation of proinflammatory cytokines in ischemia-reperfusion injury (IRI) induced acute kidney injury [59], providing evidence of the involvement of LGMN in the proinflammatory response. *In vitro*, LGMN increases the protein expression levels of Macrophage receptor with collagenous structure (MARCO) in macrophages [5], which may increase the protein expression levels of proinflammatory factors [60, 61]. Some *in vitro* studies findings suggest that LGMN may increase the expression of proinflammatory factors by increasing the expression of MARCO in macrophages. Some *in vitro* studies have shown that LGMN promotes the protein expression levels of anti-inflammatory factors in monocytes, inhibits monocyte activation and facilitates macrophage polarization toward the M2 type [48]. LGMN promotes the protein expression levels of the anti-inflammatory factors IL-10 and CD163 in primary monocytes and inhibits the protein expression levels of the proinflammatory cytokines MCP-1 and MPO. LGMN also inhibits the protein expression levels of CD14, an activation marker of monocytes. LGMN inhibits the protein expression levels of TLR2 and TLR4, which are markers of M1 macrophages, but increases the protein expression levels of MSR1, CD36, and CD163, which are markers of M2 macrophages [48]. These results identify anti-inflammatory mechanisms of LGMN in monocytes.

LGMN induces monocyte/macrophage recruitment in atherosclerosis, and in animal models of stroke, LGMN promotes inflammatory cell infiltration at sites of cerebral ischemia [62]. The aforementioned reduction of acute IRI-induced kidney injury by LGMN knockout further substantiates LGMN recruitment of monocytes/macrophages [59]. In contrast, during myocardial infarction, LGMN deficiency increases macrophage infiltration and monocyte recruitment [38]. These seemingly contradictory results may be related to the functional differences in LGMN in monocytes/macrophages in different tissues and organs. In addition, LGMN promotes monocyte/macrophage recruitment in inflammatory disease, reduce monocyte/macrophage infiltration and promote inflammation regression by promoting efferocytosis. Further studies are required to clarify the mechanism of LGMN in the occurrence, progression, and regression of CVDs.

Recently, new mechanisms of LGMN have also been discovered. Ferroptosis, an iron-dependent mode of cell death associated with lipid peroxidation [63], is involved in CVDs such as atherosclerosis, PAH, myocardial infarction, and aortic aneurysm and dissection [64–66]. Recent studies of acute kidney injury implicated LGMN in the degradation of glutathione peroxidase 4 (GPX4), a key protective factor against ferroptosis, through molecular chaperone-mediated lysosomal autophagy [59]. LGMN-mediated promotion of ferroptosis may play the same role in CVDs, which warrants further investigation.

Age is an independent risk factor for CVDs, as large and medium-sized arteries in elderly individuals exhibit changes in vascular structure and function that lead to vascular disease [67]. Lysosomes are degradation centers and signaling hubs in cells that participate in aging-related metabolism [68, 69]. LGMN is one of the key proteins involved in lysosome function, promoting the progression of aging. The protein expression levels and activation of LGMN in the brain increased with age and were associated with age-related neurodegenerative diseases [70]. The progression of neurodegenerative diseases was attenuated by pharmacologic inhibition of LGMN [71]. In CVDs, the protein expression levels of LGMN was related to aging in aortic tissue [23]. More research is needed to elucidate the underlying mechanism of LGMN in vascular aging.

Hypertension is one of the most important risk factors for CVDs. Recent studies have shown that gene expression levels of LGMN is significantly increased in CD4^+^ T cells from hypertensive patients and mice [72]. LGMN directly interacts with and promotes the degradation of tumor necrosis factor receptor-associated factor 6 (TRAF6) through chaperone-mediated autophagy, thereby inhibiting NF-κB activation and impairing regulatory T-cell (Treg) differentiation and function to prevent hypertension and its complications [72]. LGMN may be an innovative approach for treating hypertension and deserves further study.

Statins reduce cholesterol synthesis by inhibiting hydroxymethylglutaryl coenzyme A reductase of the mevalonate pathway. Statins are effective agents for the treatment of CVDs, and their pleiotropic effects on patients include increased expression of genes involved in monocyte/macrophage apoptosis, inhibition of the inflammatory response, antioxidant effects, prevention of foam cell formation, and stabilization of atherosclerotic plaques [73]. The abundance of LGMN mRNA in circulating monocytes was reduced in patients treated with atorvastatin and was further validated *in vitro* [34, 73]. Additionally, in animal experiments, atorvastatin treatment reduced the abundance of LGMN in macrophages in aortic atherosclerotic plaques [74]. The inhibitory effect of statins on the abundance and activity of LGMN further demonstrates the promise of LGMN as a new therapeutic target for CVDs.

In summary, current evidence shows that LGMN is associated with CVDs such as atherosclerosis, PAD, PAH, CAD, aortic aneurysms and dissection. In most cases, LGMN plays a harmful role in CVDs, and animal experiments show that inhibiting the function or protein expression levels of LGMN can alleviate the development of atherosclerosis, PAH, and aortic dissection. Meanwhile, in specific conditions, LGMN can also play a beneficial effect in myocardial infarction. The platelet-derived LGMN has anti-inflammatory effects and may improve outcomes in ST-elevation myocardial infarction patients [48]. And cardiac resident macrophage-derived LGMN can improve cardiac repair by promoting clearance of apoptotic cardiomyocytes after myocardial infarction through phagocytosis [52]. The underlying mechanism of LGMN in different CVDs needs further study.

## Conclusion

LGMN is a promising therapeutic target for CVDs. LGMN has both protease and non-protease functions and plays either deleterious of beneficial roles in different diseases depending on the cell source that secretes LGMN, which deserves further study. By understanding the pathogenesis and biomarker function of LGMN in CVDs, we will develop new strategies to prevent, diagnose, or treat these diseases.
